# Translational stalling at polyproline stretches is modulated by the sequence context upstream of the stall site

**DOI:** 10.1093/nar/gku768

**Published:** 2014-08-20

**Authors:** Agata L. Starosta, Jürgen Lassak, Lauri Peil, Gemma C. Atkinson, Kai Virumäe, Tanel Tenson, Jaanus Remme, Kirsten Jung, Daniel N. Wilson

**Affiliations:** 1 Gene Center and Department for Biochemistry, University of Munich, Feodor-Lynenstr. 25, 81377 Munich, Germany; 2 Department of Biology I, Microbiology, Ludwig-Maximilians-Universität München, 82152 Martinsried, Germany; 3 Wellcome Trust Centre for Cell Biology, University of Edinburgh, Edinburgh, UK; 4 Institute of Technology, University of Tartu, Tartu, Estonia; 5 Department of Molecular Biology and Laboratory for Molecular Infection Medicine Sweden (MIMS), Umeå University, Umeå, Sweden; 6 Institute of Molecular and Cell Biology, University of Tartu, Tartu, Estonia; 7 Center for integrated Protein Science Munich (CiPSM) at the University of Munich, Munich, Germany

## Abstract

The polymerization of amino acids into proteins occurs on ribosomes, with the rate influenced by the amino acids being polymerized. The imino acid proline is a poor donor and acceptor for peptide-bond formation, such that translational stalling occurs when three or more consecutive prolines (PPP) are encountered by the ribosome. In bacteria, stalling at PPP motifs is rescued by the elongation factor P (EF-P). Using SILAC mass spectrometry of *Escherichia coli* strains, we identified a subset of PPP-containing proteins for which the expression patterns remained unchanged or even appeared up-regulated in the absence of EF-P. Subsequent analysis using *in vitro* and *in vivo* reporter assays revealed that stalling at PPP motifs is influenced by the sequence context upstream of the stall site. Specifically, the presence of amino acids such as Cys and Thr preceding the stall site suppressed stalling at PPP motifs, whereas amino acids like Arg and His promoted stalling. In addition to providing fundamental insight into the mechanism of peptide-bond formation, our findings suggest how the sequence context of polyproline-containing proteins can be modulated to maximize the efficiency and yield of protein production.

## INTRODUCTION

Ribosomes translate message encoded within mRNA into an amino acid sequence. The rate of amino acid polymerization varies for each amino acid, being significantly slower for proline (Pro). Proline displays unique structure having pyrrolidine ring that spans the α-carbon (Cα) and nitrogen of the backbone. The imino rather than amino group determines Pro as a poor A-site acceptor of peptidyl moiety during peptide-bond formation (1,2), as well as poor donor when present in the P-site (3–5). Consequently, translation of stretches of three or more consecutive proline residues leads to ribosome stalling (3,6,7). The translational stalling occurs when the peptidyl-Pro-Pro-tRNA is located in the P-site (3,7) and results from slow peptide-bond formation with the Pro-tRNA located in the A-site (3). Moreover, translational stalling is also observed at diprolyl motifs (XPPZ), with the strength of stalling influenced by the nature of X and Z amino acids flanking the proline residues (3,7,8). Consistently, while polyproline stretches produce the strongest translational stalling, ribosome stalling is also observed with Asp and Ala preceding and/or with Trp, Asp, Asn and Gly following the diprolyl motif (3,7,8).

Ribosome stalling at polyproline motifs is relieved by the translation elongation factor EF-P in bacteria (3,6–8), or by the EF-P homolog, initiation factor IF5A, in eukaryotes (9). EF-P and IF5A are both modified post-translationally: EF-P is hydroxylysyl-β-lysinylated by action of YjeA (EpmA), YjeK (EpmB) and YfcM (EpmC) (10–12), whereas IF5A is hypusinylated by deoxyhypusine synthase and deoxyhypusine hydroxylase (reviewed by (13,14)). The post-translational modifications of EF-P and IF5A are critical for the ribosome stalling rescue activity of these factors (3,6,9). Strikingly, the absence of EF-P, or the modification enzymes YjeA or YjeK, leads to strong down-regulation of some but not all PPP-containing proteins *in vivo* (8), however, it remains unclear whether translation of these proteins is less dependent on modified EF-P or whether the EF-P dependence is masked by other factors *in vivo*.

Here, we employed SILAC (stable isotope labelling by amino acids in cell culture) coupled with mass spectrometry (MS) to analyse the influence of deletion of the *efp*, *yjeA*, *yjeK* or *yfcM* genes in the *Escherichia coli* BW25113 strain on the expression of PPP-containing proteins *in vivo*. As reported previously for *E. coli* strain MG1655 (8), we also found that the protein levels of many PPP-containing proteins in BW25113 remained unchanged or even up-regulated in the absence of modified EF-P. The analyses of the translation of these proteins *in vitro* revealed significantly weaker stalling efficiency at the PPP motifs of these proteins compared to PPP-containing proteins that were strongly down-regulated. A subsequent systematic analysis using *in vitro* and *in vivo* reporter assays demonstrated that the amino acid sequence upstream of the PPP motif influences the stalling efficiency, with the strongest influence being exerted by the amino acid directly preceding the PPP motif. Specifically, we demonstrated that amino acids such as Thr and Cys reduced stalling at the PPP motif whereas Arg and His strongly promoted stalling at PPP motifs. Collectively, our findings lead us to propose a model whereby the stalling at polyproline motifs is influenced by the context and thus most likely the conformation of the nascent polypeptide chain that is located within the ribosomal tunnel upstream of the stalling site.

## MATERIALS AND METHODS

### SILAC MS

SILAC (Δ*argA*, Δ*lysA*) wild-type and subsequently mutant strains were generated using P1 transduction (15) from Keio strains (16,17) as described in (12). The strains were grown in MOPS medium, supplemented with 50 μg/ml of ‘light’ arginine (R0) and lysine (K0) (Sigma) for wild-type MG1655 SILAC strain, ‘medium-heavy’ arginine (R6) and lysine (K4) (Cambridge Isotope Laboratories) for Δ*yjeA* and Δ*yfcM* deletion strains and ‘heavy’ arginine (R10) and lysine (K8) (Cambridge Isotope Laboratories) for Δ*efp* and Δ*yjeK* deletion strains. Cells were grown to mid-log and harvested by centrifugation and lysed. Cell lysates were mixed in 1:1:1 ratio (wt:Δ*yjeA*:Δ*efp* and wt:Δ*yfcM*:Δ*yjeK*) and proteins digested as described (18). Resulting peptides were fractionated as described (19) and analysed via LC-MS/MS using 240 min gradients (12). Data analysis was performed using MaxQuant v1.3.0.5 (20), with default settings for triple-SILAC analysis against *E. coli* K-12 MG1655 protein sequence database from UniProtKB (9 september 2011).

### Genome composition analyses

The tetra-peptide composition of the *E. coli* K-12 proteome (from NCBI, ftp://ftp.ncbi.nih.gov/) and expected composition was based on single amino acid frequencies. The expected frequency of a XPPP motifs was calculated using (p^2^x)g, where p is the fraction of proline in the genome, x is the fraction of the amino acid X and g is the genome size in amino acids.

### *In vitro* coupled transcription-translation

Templates for genes encoding LepA, NlpD, Agp, NudC, ClsA and YcgL were prepared as PCR product containing T7 promoter. *nlpD* and *lepA* were additionally cloned into pET21b (Merck) using NdeI, SacI restriction sites. Mutagenesis of *nlpD* and *lepA* was done using Xtreme Hot KOD Start DNA Polymerase (Merck). Primers, plasmids and strains used in this study are listed in Supplementary Table S1. *In vitro* translation reactions were performed using the PURExpress Δaa ΔtRNA kit (New England Biolabs), in the presence or absence of EF-P as described previously (6). Where indicated, amino acid mixes (final concentration of 0.3 mM each amino acid, pH 7.4) lacking either glutamine or threonine were used. Modified EF-P was prepared as described (6). The progress of reactions was monitored either by incorporation of [^35^S]-methionine (SDS-PAGE) or by toeprinting. We observe an unidentified 10 kDa band in the sodium dodecyl sulphate-polyacrylamide gel electrophoresis (SDS-PAGE) experiments, which is also observed when mixing translation extract with [^35^S]-methionine in the absence of mRNA template (21). Since the intensity of the 10 kDa product does not increase over time, we have used this band for normalization for the loading of the lanes and thus express the amount of full-length (FL) products relative to the 10 kDa band.

### Toeprinting assay

*In vitro* translation reactions using the PURExpress Δaa ΔtRNA kit (New England Biolabs), in the presence of primer (Supplementary Table S1) labelled with 6-FAM at 5’ end, were carried out for 30 min at 37°C, followed by addition of 100 units of reverse transcriptase (RT) Superscript II (Life Technologies) and dNTP mix to the final concentration of 400 μM. RT reaction was carried out for additional 30 min at 37°C. Products of the reactions were purified using Nucleotide Removal Kit (Qiagen) and lyophilized. Pellets were resuspended in a formamide loading dye solution and applied on the 6% urea-acrylamide gel. Fluorescence of samples was detected using Typhoon FLA 9500 scanner. Intensity of stalled bands was acquired using ImageJ. To correlate the toeprint bands with the site of ribosome stalling, sequencing reactions were performed on the NlpD template using coupled transcriptions-translation (PURExpress kit) without addition of amino acids and tRNA (Supplemental Figure S1). Reactions were supplemented with 6-FAM-labelled primer used for toe-printing and incubated for 30 min, followed by addition of 100 Units of Superscript III (Life Technologies) and ddNTP/dNTP mix (Roche) to the final concentration of 4 mM/400 μM, respectively. Reactions were then further incubated for 30 min at 37°C and the products of were purified and subjected to electrophoresis as described for the toeprinting reactions.

### β-Galactosidase assays

Cells producing LacZ hybrids under control of the *cadC* promoter were grown in Lysogeny Broth to exponential growth phase (OD_600_ 0.3–0.5). β-Galactosidase activities were then determined as described by (6) for at least three independent experiments. Primers, plasmids and strains used in this study are listed in Supplementary Table S1.

## RESULTS

### Translational stalling of up-regulated polyproline-containing proteins

**Figure 1.**
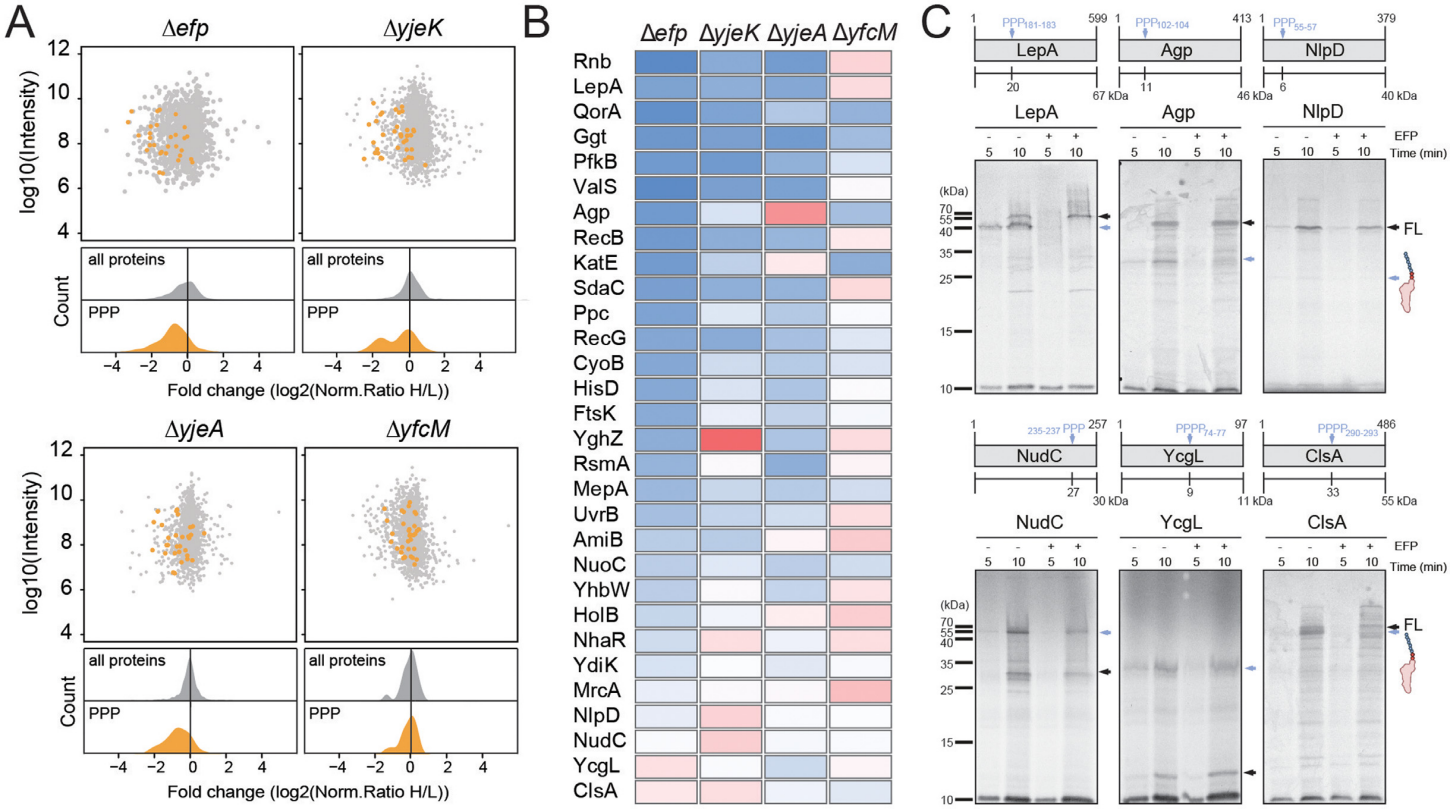
Proteomic analysis of BW25113 strains lacking *efp*, *epmA, epmB and epmC*. (**A**) Scatter and density plots of the inverted normalized H/L ratios (log2-transformed) relative to the summed-up protein intensity for a SILAC data from the Δ*efp*, Δ*yjeK*, Δ*yjeA and* Δ*yfcM* for all proteins (grey) and PPP-containing proteins (orange). (**B**) Heat map representation of the 30 identified PPP-containing proteins that were up- (red) and down- (blue) regulated in the Δ*efp*, Δ*yjeK*, Δ*yjeA* and Δ*yfcM* strains relative to wild-type strain. (**C**) *In vitro* translation time course of selected PPP-containing protein. Autoradiographs of SDS-PAGE and analysis of [^35^S]Met-labelled LepA, Agp, NlpD, NudC, YcgL and ClsA. All reactions were performed in the absence (−) and presence (+) of active EF-P. The position of FL protein and intermediate peptidyl-tRNA indicated with black and blue arrows, respectively. Schematic representations of each protein are included, with the site of the PPP motif indicated by a blue arrow.

To reassess the influence of EF-P on production of polyproline-containing proteins *in vivo*, we employed SILAC and MS to analyse protein expression levels in wild-type *E. coli* strain compared to the same strain lacking either the gene for EF-P (Δ*efp*) or one of the *E. coli* EF-P modification enzymes (Δ*yjeK*, Δ*yjeA* or Δ*yfcM*). In contrast to our previous study using *E. coli* strain MG1655 (8), here we used BW25113 to ascertain whether we observe similar trends with a different *E. coli* strain. We identified a total number of ∼1300 proteins with the normalized protein ratios distributed around log2 = 0 common to each dataset, indicating little or no change in protein expression pattern for the majority of proteins when comparing the wild-type and knock-out strains (Figure 1A). In contrast, we identified 30 of the possible 96 polyproline-containing proteins in BW25113, most of which exhibited a significant down-regulation in the Δ*efp*, Δ*yjeK*, Δ*yjeA* (Figure 1A), but not the Δ*yfcM* strain (Figure 1B), consistent with the finding that hydroxylation of EF-P by YfcM is dispensable for EF-P activity (3,6). Amongst the most down-regulated proteins were the ribonuclease II Rnb, the translation factor LepA and the quinone oxidoreductase A QorA (Figure 1B). Surprisingly, some polyproline-containing proteins showed only minor down-regulation or even exhibited up-regulation in the absence of active EF-P, for example, cardiolipin synthase (ClsA), NADH pyrophosphatase (NudC), murein hydrolase activator (NlpD) and YcgL, a protein of unknown function. A similar up-regulation of polyproline-containing proteins in the absence of active EF-P was observed previously in *E. coli* MG1655 strain, with the largest up-regulation observed for ClsA, NlpD, DppF and Agp (8). Up-regulation in expression of these proteins does not necessarily indicate that translation of these proteins is independent of EF-P since any EF-P dependence *in vivo* may be masked by other factors, such as increased transcription or decreased mRNA degradation. Therefore, to directly assess the EF-P dependence of translation of up-regulated protein, we employed an *in vitro* translation system reconstituted from purified components to analyse the translation of five up-regulated proteins, Agp, ClsA, NlpD, NudC, YcgL, in the presence and absence of active EF-P (Figure 1C). As a positive control, translation of the down-regulated LepA protein was included, which was previously shown to require the presence of active EF-P for efficient translation *in vitro* (7). Translation of LepA in the absence of EF-P led to appearance of two prominent bands; a ∼40 kDa peptidyl-tRNA band reflecting the mass of the LepA polypeptide translated up to the PPP motif (∼20 kDa) but remaining attached to tRNA (∼20 kD), and a 67kDa band corresponding to the FL LepA protein (Figure 1C). As expected, the presence of active EF-P rescues ribosomes stalled at the PPP motif (3,6) and leads to loss of the peptidyl-tRNA band (Figure 1C). Similar results were observed for Agp, and ClsA with peptidyl-tRNA bands observed in the absence of EF-P but absent in the presence of EF-P, however, the EF-P rescue was less dramatic for NudC and YcgL. Overall, these results indicate that despite being up-regulated in the absence of active EF-P *in vivo*, these polyproline-containing proteins nevertheless require active EF-P for efficient translation. We note however that compared to translation of LepA, which produced a distinct peptidyl-tRNA band after 5 min, the peptidyl-tRNA band for the Agp, NudC, YcgL and ClsA was generally weaker in intensity (Figure 1C), suggesting that ribosome stalling during translation of these proteins may be less efficient. This was exemplified by translation of NlpD where no obvious peptidyl-tRNA band was observed in the absence of EF-P, despite the efficient translation of the FL polyproline-containing NlpD protein (Figure 1C).

### The context of PPP motifs influences the inefficiency of ribosome stalling

**Figure 2.**
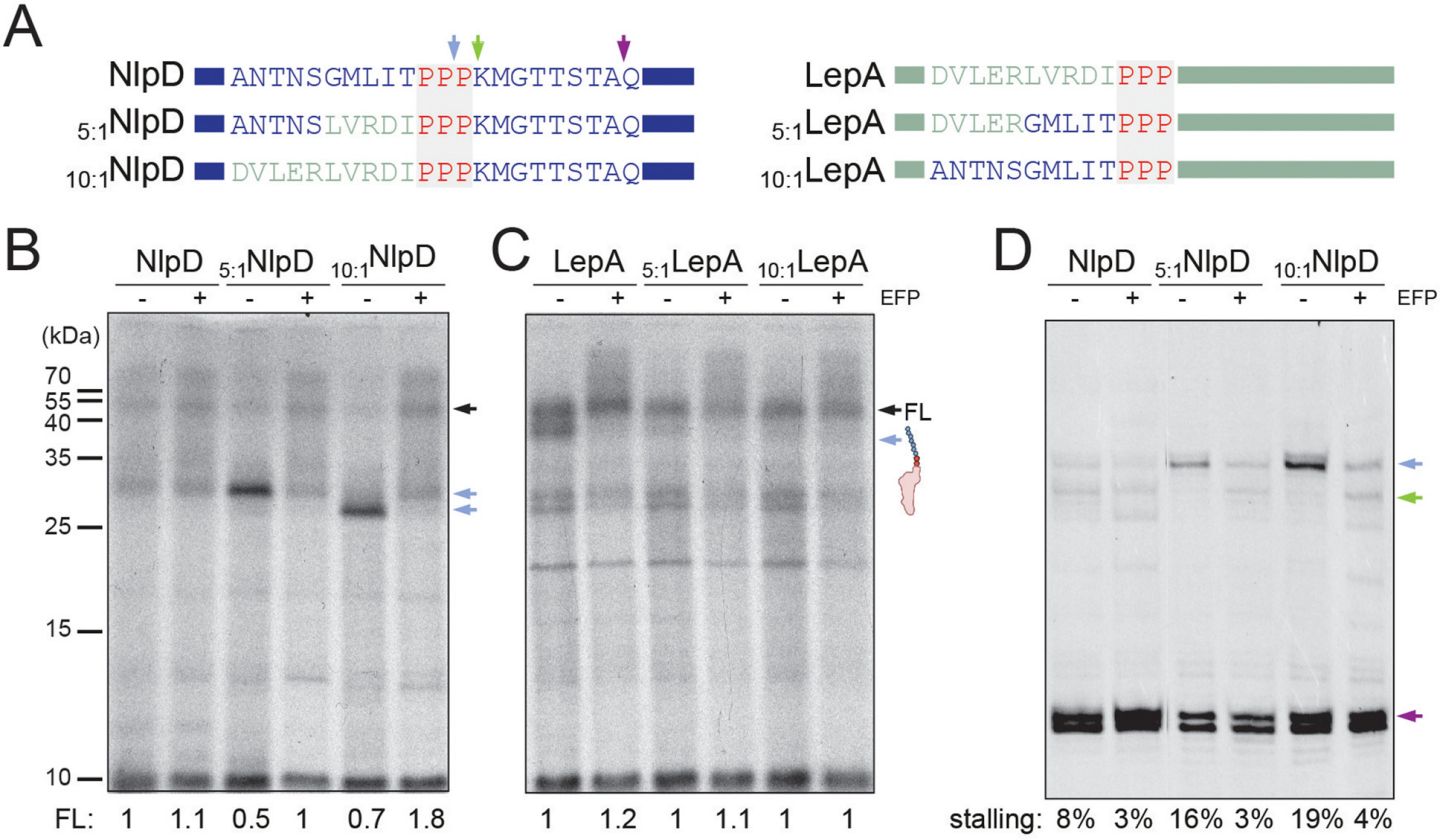
The influence of the PPP-context on efficiency of the stalling. (**A**) Schematic representations of chimeras constructed between NlpD and LepA with sites of stalling observed in the absence of EF-P (green and blue arrows) or in the absence of the amino acid glutamine (purple arrow). (**B,C**) Autoradiograms of SDS-PAGE of *in vitro* translation reactions of (B) wild-type NlpD, _5:1_NlpD and _10:1_NlpD chimeras and (C) wild-type LepA, _5:1_LepA and _10:1_LepA chimeras. The position of the FL protein and peptidyl-tRNA is indicated with black and blue arrows, respectively. In (B) and (C), the normalized values for the FL product are indicated for each respective lane with wild-type FL product for NlpD and LepA assigned as 1. (**D**) Toeprinting analyses of ribosome stalling during translation of NlpD chimeras. Stalling at peptidyl-Pro55-Pro56-tRNA in the P-site and Pro57-tRNA in the A-site (blue arrow) and with pep-PPP-tRNA in the P-site and Lys58-tRNA in the A-site (green arrow) is also shown in (A). The lack of glutamine arrests ribosomes at Q66 (violet arrow). Stalling efficiency is calculated as a percentage of stalling at PP/P and PPP/K relative to summed up intensities of PP/P, PPP/K and Q. All reactions were performed in the absence (−) and presence (+) of active EF-P.

To investigate why ribosome stalling occurs efficiently at the PPP motif present in LepA but inefficiently at the PPP motif present in NlpD, we constructed chimeras between LepA and NlpD, namely, with five or ten of the amino acids preceding the PPP motif substituted between NlpD and LepA. Thus, the chimera _5:1_NlpD and _10:1_NlpD contained five and ten amino acids, respectively, from LepA located directly before the PPP motif of NlpD, whereas _5:1_LepA and _10:1_LepA contained five and ten amino acids, respectively, from NlpD placed directly before the PPP motif of LepA (Figure 2A). As before, translation of the NlpD, LepA and the chimeras was performed *in vitro* in the presence and absence of EF-P (Figure 2B and C). In agreement with our previous observations, *in vitro* translation of NlpD in the absence of EF-P showed no obvious peptidyl-tRNA band, however, translation of _5:1_NlpD and _10:1_NlpD led to the appearance of a distinct band at the expected size of the peptidyl-tRNA (26 kDa) (Figure 2B). Consistently, this peptidyl-tRNA band was absent when EF-P was present (Figure 2B). Conversely, while translation of LepA led to the presence of a peptidyl-tRNA band (∼40 kDa) in the absence of EF-P, no obvious peptidyl-tRNA band was observed during translation of the _5:1_LepA and _10:1_LepA chimeras (Figure 2C).

We also employed the toeprinting assay to monitor ribosome stalling during translation of NlpD and the NlpD chimeras (Figure 2D), as used previously to monitor stalling at PPP motifs (7). The toeprinting assay uses reverse transcription to monitor the exact distance between the stalled ribosome and the downstream primer annealing site on the mRNA (22). Moreover, since there are no glutamines (Q) encoded between the N-terminus of NlpD and the PPP motif (positions 55–57), we could perform translation reactions with an amino acid mix lacking glutamine so as to monitor ribosomes that do not stall at the PPP motif by trapping them on the downstream glutamine codons (positions 66–67) (Figure 2A and D). This allowed us to quantify the relative strength of stalling at the PPP motifs in comparison to the total translation of the mRNA (Figure 2D). Consistent with the *in vitro* translation results (Figure 2B), weak ribosome stalling (8%) was detected during translation of NlpD. Two main sites of stalling were observed, namely, the first corresponding to peptidyl-Pro55-Pro56-tRNA in a P-site and Pro57-tRNA in the A-site, and a second located one codon downstream, i.e. with peptidyl-PPP-tRNA in the P-site and Lys-tRNA in the A-site. In contrast, only a single predominant stall site (16–19%) was observed for the NlpD chimeras, which corresponded with the first site, suggesting that peptide-bond formation is inefficient between the peptidyl-Pro55-Pro56-tRNA in a P-site and Pro57-tRNA in the A-site, as noted previously for stalling at PPP motifs (7). As expected, the presence of active EF-P alleviated stalling at the first site, allowing stalling at the second downstream site to be observed (Figure 2D). We note that the ribosome stalling was transient and, even in the absence of EF-P, ribosomes eventually catalyzed peptide-bond formation between the P- and A-site substrates leading to accumulation of ribosomes trapped on the downstream glutamine codon. Collectively, our findings suggest that the nature of the amino acids preceding the PPP motif can have a marked influence the efficiency of stalling.

### The amino acids preceding the PPP motif influence the stalling efficiency

**Figure 3.**
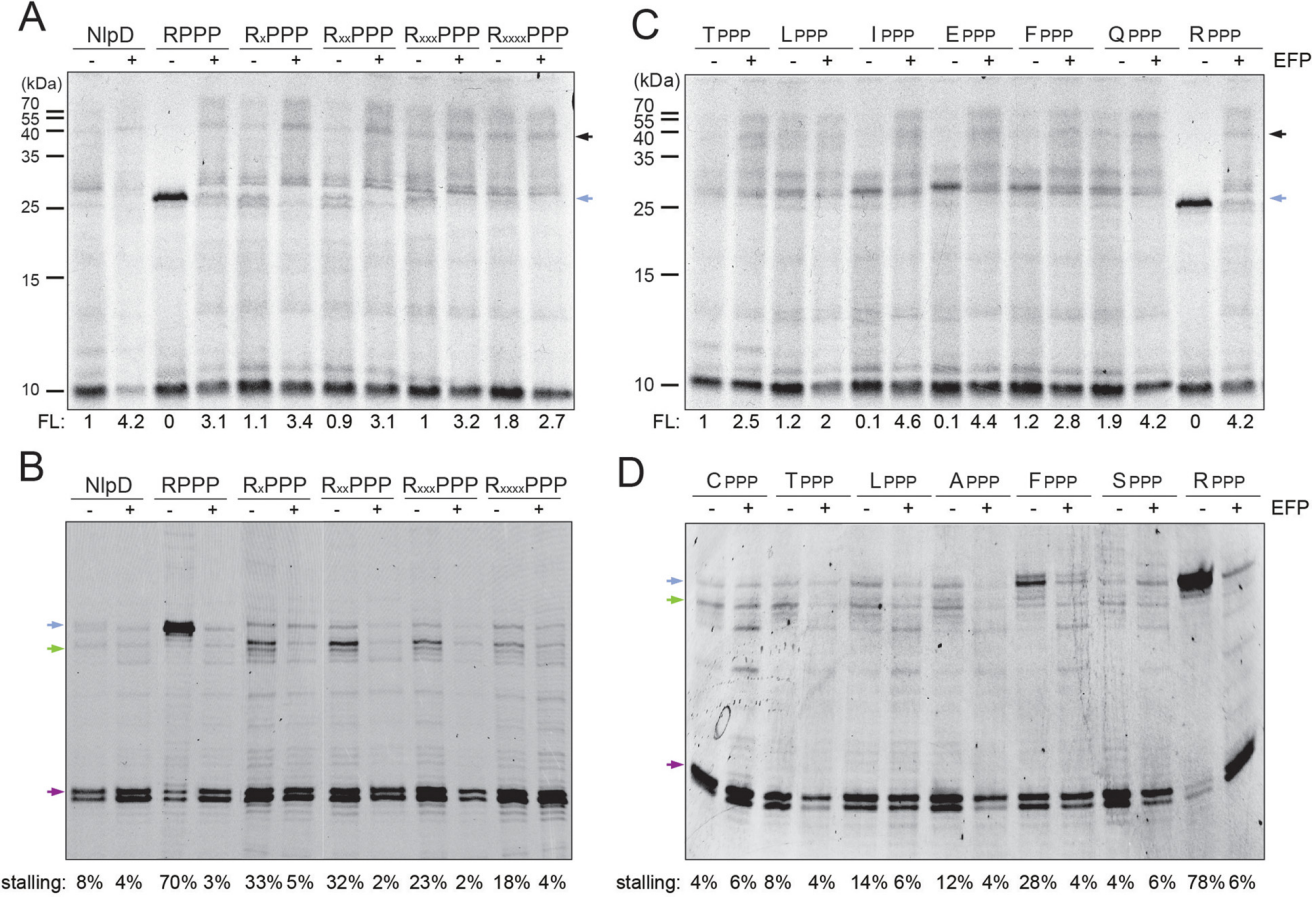
The influence of the amino acids preceding the PPP motif on stalling efficiency. (**A,B**) *In vitro* translation of arginine-scanning mutants of NlpD variants monitored by (A) incorporation of [^35^S]Met and (B) toeprinting. (**C,D**) *In vitro* translation of NlpD variants with single-substituted amino acids T, L, I, F, A, S, E, Q at positions directly preceding the PPP motif, monitored by (C) incorporation of [^35^S]Met and (D) toeprinting. In (A) and (C), the position of the FL protein and peptidyl-tRNA is indicated with black and blue arrows, respectively. In (B) and (C), the normalized values for the FL product are indicated for each respective lane with wild-type FL product for NlpD and LepA assigned as 1. In (B) and (D), stalling with peptidyl-Pro55-Pro56-tRNA in the P-site and Pro57-tRNA in the A-site is shown with a blue arrow, and pep-PPP-tRNA in the P-site and Lys58-tRNA in the A-site with a green arrow. Stalling efficiency was calculated as in Figure 2D. All reactions were performed in the absence (−) and presence (+) of active EF-P.

Next, we investigated which amino ACID position(s) preceding the PPP motif are responsible for the reduced stalling efficiency of NlpD. We focused on the first five amino acids preceding the PPP motif, GMLIT, since substitution of these by LVRDI from LepA was sufficient to induce significant stalling (Figure 2B and D). We have previously demonstrated that the tripeptide motif APP induces stalling, whereas RPP does not (8), therefore to minimize XPP effects on the XPPP context we decided to initially perform arginine, rather than alanine, scanning mutagenesis on the GMLIT sequence of NlpD (Figure 3A). Surprisingly, mutation of Thr to Arg (RPPP) in the −1 position relative to the PPP motif of NlpD was sufficient to induce very strong stalling, as apparent by the intense peptidyl-tRNA band observed during *in vitro* translation reactions in the absence of EF-P (Figure 3A). Although arginine mutations at the −2 to −5 positions (GMLI) also produced increased stalling compared to the wild-type NlpD sequence, the stalling was weaker than the arginine mutation at the −1 position (Figure 3A). Furthermore, the presence of EF-P rescued the stalling regardless of the strength of the stall (Figure 3A). We also performed toeprinting on NlpD and the NlpD arginine mutants to better quantify the EFFICIENCY of stalling as well as determine the exact site of stalling (Figure 3B). In contrast to the weak stalling observed for wild-type NlpD (8%), the −1 Arg mutation led to very strong stalling (70%) with the peptidyl-Pro55-Pro56-tRNA in the P-site and Pro57-tRNA in the A-site (Figure 3B). In agreement with the *in vitro* translation assays (Figure 3A), arginine mutations at the −2 to −5 positions (GMLI) also increased stalling compared to the wild-type NlpD sequence, with the strength of stalling declining from 33% to 18% as the arginine substitutions were moved from the −2 to −5 position (Figure 3B). Unexpectedly, the stalling site was shifted by one codon upstream when Arg was in the −1 position as compared with the −2 to −5 positions, i.e. in the latter constructs stalling occurred with peptidyl-PPP-tRNA in the P-site and Lys58-tRNA in the A-site (Figure 3B). This latter observation suggests that THE context of the PPP motif can not only influence the strength of stalling but also modulate the exact site where stalling occurs. In summary, our findings indicate that the -1 position directly preceding the PPP motif exerts the strongest influence on the efficiency of stalling at the PPP motif, and that the −2 to −5 positions also contribute, with the contribution progressively decreasing as the distance from the PPP motif increases.

### The nature of the amino acid preceding the PPP motif influences stalling

Of the 96 polyproline-containing proteins in *E. coli*, threonine directly before the proline cluster (i.e. TPPP as in NlpD) is over-represented, being present 17 times compared to the expected five times (Supplementary Figure S2). Another highly over-represented XPPP motif is LPPP, which has 22 occurrences compared to the expected 11. Curiously, we noted that the proteins that exhibited only modest down-regulation (or even up-regulation) in the absence of EF-P, namely ClsA, YcgL, NudC, NlpD and MrcA (Figure 1B), all contain LPPP or TPPP motifs. In contrast, RPPP occurs twice compared to the expected six times (Supplementary Figure S2), but was only identified once in our analysis, namely for RecG, which exhibited a strong down-regulation in the absence of active EF-P (Figure 1B). Therefore, to assess how the nature of the amino acid in the −1 position influences stalling at PPP motifs, we performed *in vitro* translation (Figure 3C) and toeprinting (Figure 3D) assays as before for NlpD, but with mutation of the wild-type NlpD TPPP motif to LPPP, APPP, CPPP, EPPP, FPPP, IPPP, QPPP and RPPP. Collectively, the results indicated weak stalling at LPPP, CPPP, APPP and SPPP (similar levels to the wild-type TPPP motif of NlpD) and intermediate stalling at EPPP, FPPP, IPPP and QPPP, however really strong levels of stalling were only observed for RPPP (Figure 3C and D).

**Figure 4.**
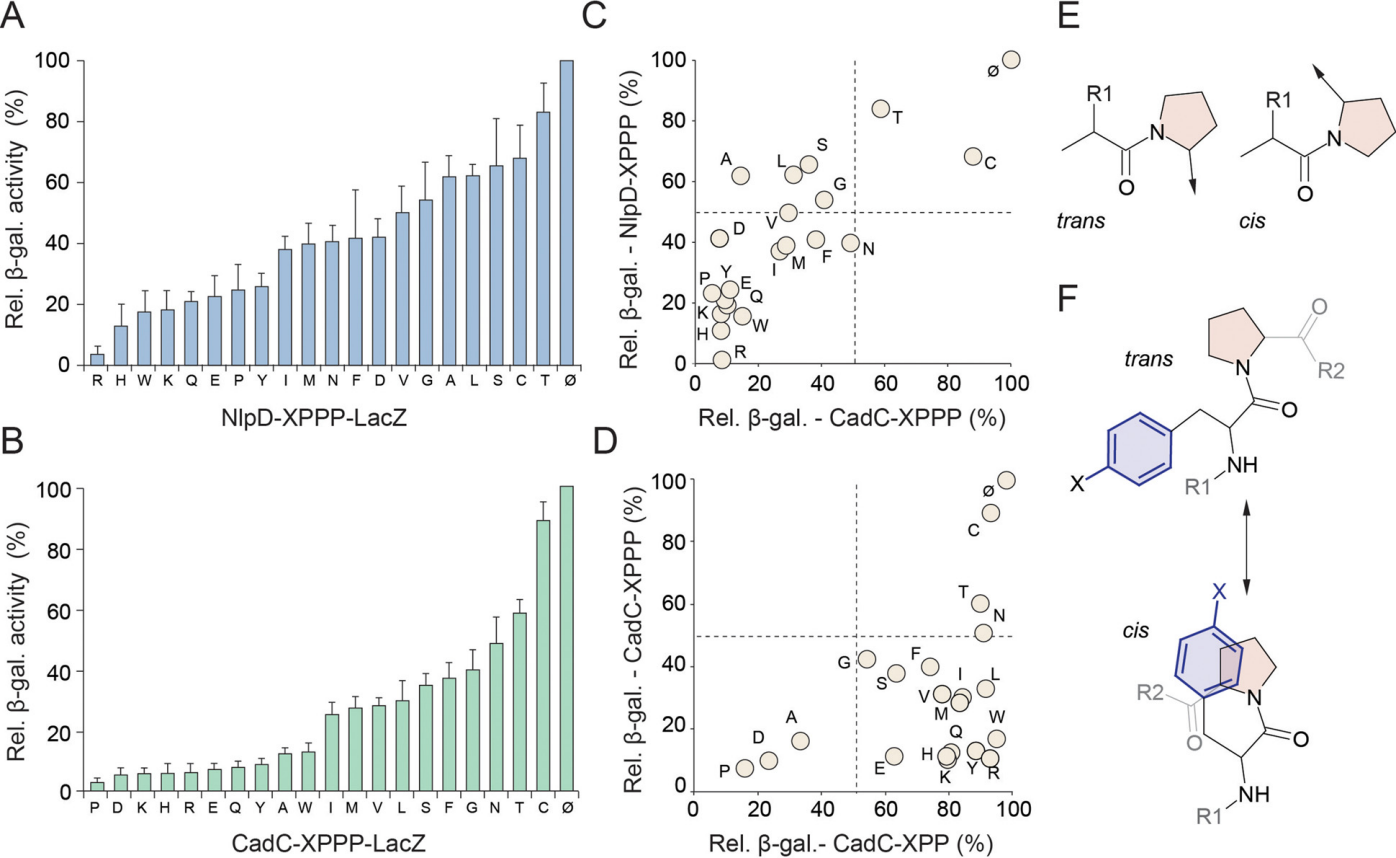
Differential influence of amino acids at position -1 on efficiency of PPP-stalling. (**A,B**) Relative β-galactosidase activities of (A) NlpD-XPPP and (B) CadC-XPPP fusions with LacZ, where X represents one of the 20 proteinogenic amino acids, were determined by monitoring the β-galactosidase activities of the LacZ fusions in wild-type *E. coli* strains relative to the Δ*efp* strain. Values were normalized relative to a control LacZ construct lacking the PPP motif (no stalling) which was assigned as 100%. (**C**) Scatter plot of relative β-galactosidase activities of NlpD-XPPP-LacZ relative to CadC-XPPP-LacZ constructs. (**D**) Scatter plot of relative β-galactosidase activities of CadC-XPPP-LacZ relative to CadC-XPP-LacZ fusions (8). (**E**) Chemical structure of proline in *trans* and *cis* conformation. (**F**) Possible *trans* and *cis* conformations of peptide containing aromatic amino acids followed by proline.

To more systematically and quantitatively assess the influence of the nature of the −1 position on stalling at PPP motifs, we fused the first 57 amino acids of NlpD (up to and including the TPPP motif) without a stop codon to LacZ. We then substituted Thr54 of NlpD with the remaining 19 proteinogenic amino acids and the efficiency of the stalling of each NlpD-LacZ fusion construct was monitored *in vivo* by comparing the β-galactosidase activity in the Δ*efp* and wild-type *E. coli* (*efp^+^*) strains (Figure 4A). Consistent with our *in vitro* data (Figure 3), we also observed a strong influence of the nature of the amino acid in the −1 position on ribosomal stalling, with a gradient of effects ranging from the strongest stalling with RPPP to the weakest stalling occurring with TPPP (Figure 4A). In order to evaluate whether the hierarchy of the effect of the −1 position on stalling at the PPP motif in NlpD is similar for other proteins, we repeated the experiment using the N-terminal context of the *E. coli* pH stress sensor CadC followed by XPPP and LacZ (6). As before, we generated CadC-XPPP-fusion where the −1 position (X) of the PPP motif contained each of the 20 amino acids and then monitored the β-galactosidase activity of each construct *in vivo* in the presence and absence of EF-P (Figure 4B). Similar to the NlpD-XPPP-LacZ results, a gradient of effects of the −1 position was also observed using the CadC-XPPP-LacZ construct, however the hierarchy differed slightly, such that PPPP was observed to induce the strongest stalling and the weakest stalling was observed for CPPP (Figure 4B). In our previous work, APP was identified as a strong staller when using the CadC-LacZ reporter (8), and consistently, in this study we also see that APP is a good staller in the context of APPP and the CadC-APPP-LacZ reporter (Figure 4B). In contrast, APP is observed as a weak staller in the context of APPP and the NlpD-APPP-LacZ reporter (Figure 4A). A likely explanation for this is that the context upstream of the XPPP motif can influence the stalling efficiency, as we observed when analysing mutations in the −2 to −5 positions of NlpD (Figure 3A and B). While we cannot exclude that differences in stability of the CadC and NlpD reporter mRNAs or proteins contribute to the differences in the exact hierarchies, we nevertheless observe a good overall correlation between the two datasets, such that weak stalling was observed for CPPP and TPPP, whereas EPPP, HPPP, KPPP, QPPP, RPPP, YPPP, WPPP and PPPP clustered together as strong stalling motifs (Figure 4C). In contrast, a poor correlation was observed between the strength of stalling at XPPP motifs compared to our previous analysis (8) of stalling at XPP motifs using the same CadC context (Figure 4D). This is not unexpected since we demonstrated that the upstream context of the XPPPZ motif can influence the exact site of stalling, shifting it by one codon downstream from XPP/PZ to XPPP/Z (Figure 3B), whereas stalling at XPP/Y motifs already occurs after the last proline residue (7,8).

## DISCUSSION

Our analysis of the changes in expression levels of proteins in *E. coli* K12 strains BW25113 (Figure 1A) and MG1655 (8) revealed an expected down-regulation for most polyproline-containing proteins in the absence of EF-P or the EF-P modification enzymes YjeA and YjeK. However, for some polyproline-containing proteins the expression level did not change or even increased (Figure 1B), as noted previously (8). Using a series of *in vitro* and *in vivo* translation reporters, we revealed that the context upstream of the polyproline motif can influence the efficiency of stalling and therefore modulate the dependence of the protein expression on EF-P. Specifically, PPP motifs preceded by amino acids C and T, and also G, L, S, displayed reduced stalling efficiency (Figures 3C,D and 4C). To ascertain whether this trend was also observed *in vivo*, we performed a re-analysis of the proteomics data presented here for BW25113, as well as of the previous study for MG1655 (8). Consistent with our *in vitro* data, computational removal of the polyproline-containing proteins with PPP motifs preceded by G, L, S, C and T led to a much larger down-regulation in the distribution of PPP-containing proteins for the Δ*efp*, Δ*yjeA*, Δ*yjeK*, but not the Δ*yfcM* strain (Supplementary Figure S3). Unfortunately, the same analysis cannot be made for the strong stalling XPPP motifs since they are highly underrepresented in the *E. coli* proteome (Supplementary Figure S2) and very few were detected in the proteomic studies. This is particularly true for the aromatic XPPP motifs (F, H, W, Y), which are expected a total of 10 times, but are found only twice, namely QorA (YPPP) and AdrA (HPPP). Only QorA was identified in the proteomic analyses, and in both cases was strongly down-regulated (Figure 1B) (8).

**Figure 5.**
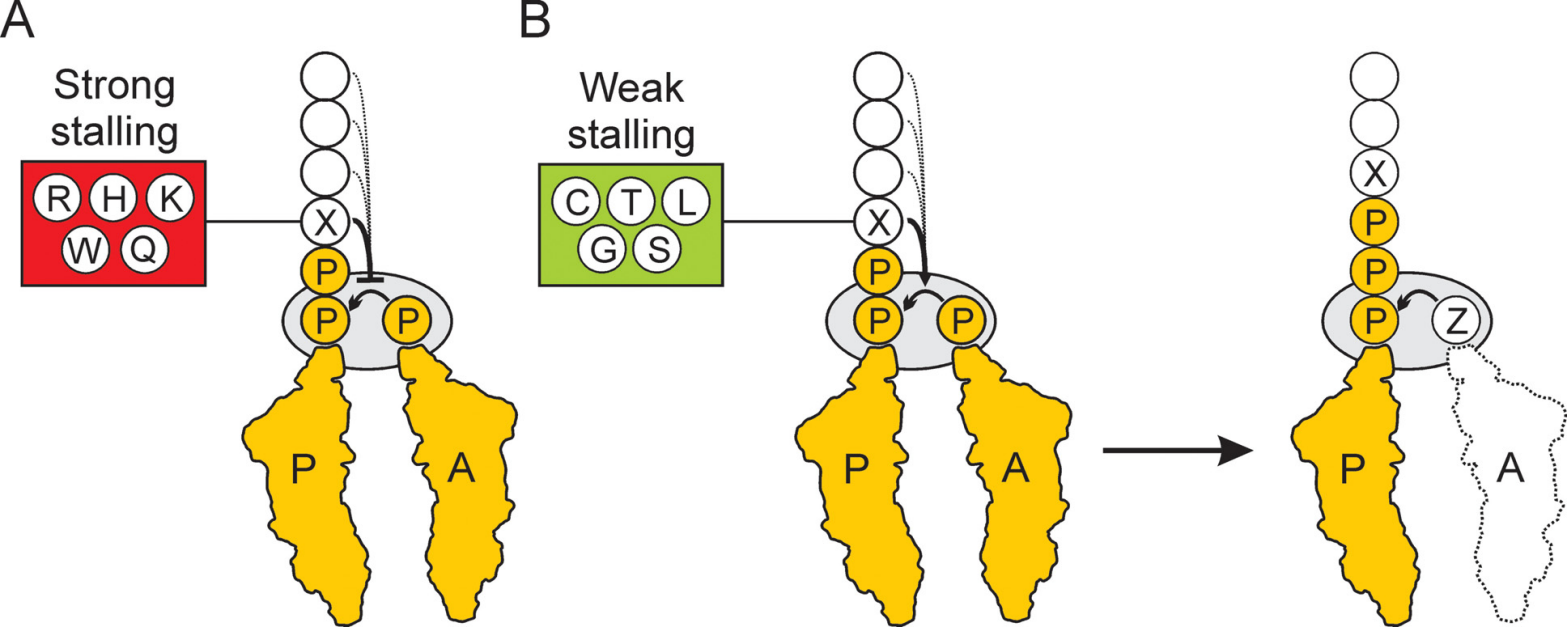
Model for ribosome stalling at XPPPZ motifs. (**A**) The amino acids R, H, K, Q or Q located at position X preceding the PPP motif strongly decrease the rate of peptide-bond formation between the Peptidyl-Pro-Pro-tRNA in the P-site and Pro-tRNA in the A-site. The efficiency of stalling can be further modulated by the upstream amino acids (dashed line). (**B**) The amino acids C, T, L, G or S located at position X preceding the PPP motif promote peptide-bond formation between the Peptidyl-X-Pro-Pro-tRNA in the P-site and Pro-tRNA in the A-site. Subsequent stalling then occurs with Peptidyl-X-Pro-Pro-Pro-tRNA in the P-site and Z-tRNA in the A-site. In this case, the efficiency of stalling can be modulated by the nature of the Z amino acid (1–3,7,8).

Compared to other amino acids, proline can readily adopt both *cis* and *trans* conformations (Figure 4E) and the propensity to adopt a *cis* or *trans* conformation within a peptide or protein is influenced by the context of the proline residue(s) (23,24). Within model peptides as well as proteins in the protein databank (PDB), proline–proline, aromatic–proline and proline–aromatic sequences have the highest propensity to adopt *cis*-prolyl amide bonds (25–27). Subsequent studies suggested that large aromatic residue preceding proline can promote *cis*-proline conformations by establishing interaction between the π aromatic face of the aromatic side chains and the polarized C-H bonds of proline (Figure 4F) (23,24). However, such studies are performed in solution and therefore it is unclear how they relate to the isomerisation of proline residues within peptides attached to tRNAs and within the confines of the ribosomal tunnel. Although many aromatic residues were present within the cluster of strong stalling XPPP motifs, for example, HPPP, WPPP and YPPP (Figure 4), we also observe strong stalling when many non-aromatic amino acids precede the PPP motif, namely amino acids with relatively long side chains (R, K, D, E and Q). Thus we favour a model whereby the physical and/or chemical properties of particular amino acids preceding the PPP stalling motif influence the conformation of the proline-containing nascent polypeptide chain in a manner that is unfavourable for peptide-bond formation, for example with R, H, K, W or Q (Figure 5A). Moreover, our results revealing the presence of a different hierarchy of stalling efficiency for XPPP motifs in NlpD and CadC (Figure 4A and B) indicate that the upstream context of the XPPP stalling motif also contributes to modulating the efficiency of stalling (Figure 5A). In contrast, we also identified a subset of amino acids that presumably promote conformations of the proline-containing nascent polypeptide chain that are favourable for peptide-bond formation, for example with C, T, L, G and S (Figure 5B). In this case, we observe that while stalling at the XPP/PZ motif is reduced, subsequent stalling at the downstream XPPP/Z motif then ensues (Figure 5B), with the strength of the stalling being influenced by the amino acid attached to the incoming A-tRNA (1–3,7,8).

The ability of amino acid sequence of the polypeptide chain within the ribosomal tunnel to modulate the efficiency of peptide-bond formation and thereby induce translational arrest has been reported for a wide variety of stalling leader peptides (28), with well-characterized examples including the SecM (29) and Erm-type leader peptides (30–32). In these specific examples the nature of the amino acid in the −2 position (i.e. equivalent to the X position in the XPP/P stalling motif) was also shown to be critical for stalling. In SecM, the −2 position is a conserved arginine (R163) that when mutated abolishes the translation arrest (29,33). Similarly, the −2 position is critical for drug-dependent stalling induced by the ErmAL and ErmCL leader peptides (34,35). ErmCL contains phenylalanine (F7) in the −2 position and stalls the ribosome regardless of the nature of the A-site aa-tRNA (restrictive) (34), whereas ErmAL contains alanine (A6) in the −2 position and the nature of the A-site aa-tRNA dramatically influences stalling (selective). In fact the hierarchy of the influence of the A-site amino acid on stalling bears some resemblance to that observed at the XPPP motifs, namely, that strong stalling occurs when positively charged amino acids, e.g. W, R and K, were in the A-site, but inefficient stalling was observed with more hydrophobic amino acids such as C, F and M (35). Strikingly, swapping the amino acid in the −2 position between ErmAL and ErmCL appeared to swap the specificity, such that ErmAL became restrictive and ErmAL selective, thus illustrating the ability of the amino acid in the −2 position to modulate peptide-bond formation during drug-dependent stalling, analogous to our findings during polyproline-dependent stalling. Future studies addressing the structures of stalled ribosomes will be necessary to provide insight into how the nascent polypeptide within the tunnel can influence peptide-bond formation at the peptidyl transferase centre of the ribosome.

## SUPPLEMENTARY DATA

Supplementary Data are available at NAR Online.

SUPPLEMENTARY DATA
