# Supplementary material for: Translational stalling at polyproline stretches is modulated by the sequence context upstream of the stall site
Source: Nucleic Acids Res. 2014 Aug 20;42(16):10711–9. doi: 10.1093/nar/gku768 (PMC4176338; doi:10.1093/nar/gku768)
Supplement: SUPPLEMENTARY DATA [file supp_42_16_10711__index.html]

Translational stalling at polyproline stretches is modulated by the sequence context upstream of the stall site — Translational stalling at polyproline stretches is modulated by the sequence context upstream of the stall site — SUPPLEMENTARY DATA 

# Translational stalling at polyproline stretches is modulated by the sequence context upstream of the stall site

## SUPPLEMENTARY DATA

**Files in this Data Supplement:**

- SUPPLEMENTARY DATA
