# Supplementary material for: Translational stalling at polyproline stretches is modulated by the sequence context upstream of the stall site
Source: Nucleic Acids Res. 2014 Aug 20;42(16):10711–9. doi: 10.1093/nar/gku768 (PMC4176338; doi:10.1093/nar/gku768)
Supplement: SUPPLEMENTARY DATA [file supp_gku768_nar-01963-v-2014-File007.pdf]

SUPPLEMENTARY DATA

Supplementary Figures

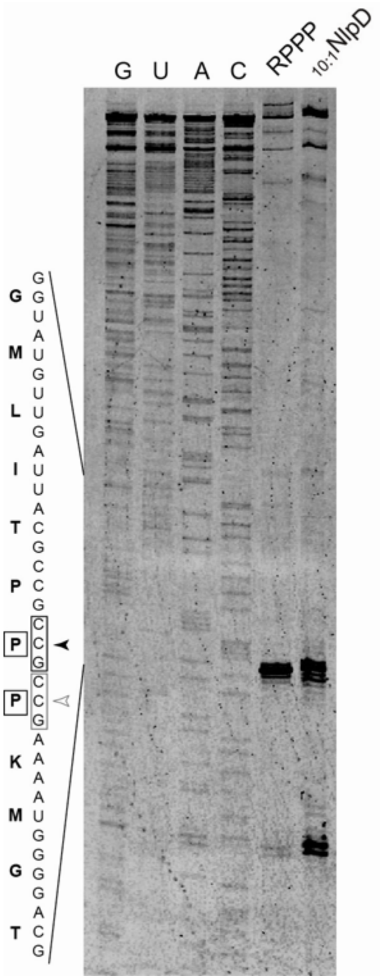

Supplementary Figure S1. Assignment of ribosome stalling sites in the toeprinting reactions for the RPPP and chimeric $_{10:1}$NlpD constructs.

Sequencing reactions were used to determine the site of ribosome stalling on the mRNAs of the RPPP and chimeric $_{10:1}$NlpD constructs. The black and white arrows indicate the codons present in the P- and A-sites, respectively. The toeprinting reactions were performed in absence of EF-P and absence of the amino acid glutamine.

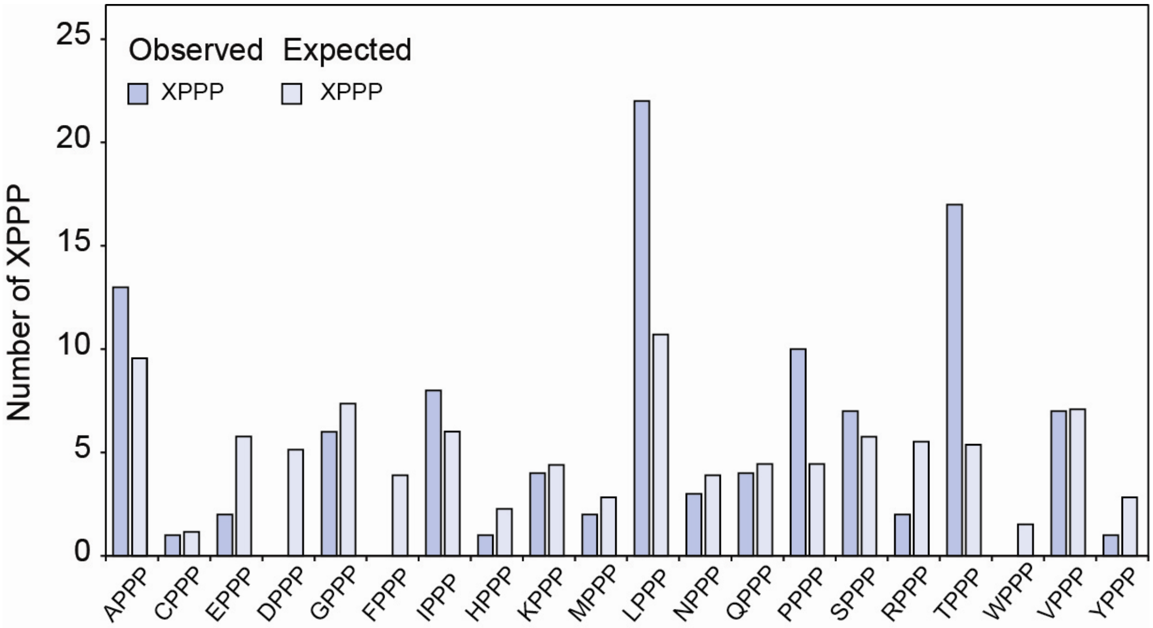

Supplementary Figure S2. Occurrence of XPPP motifs in the *E. coli* proteome

Bar graph showing the expected (light blue) and observed (dark blue) frequencies of occurrence for different XPPP motifs within proteins encoded in the *E. coli* genome

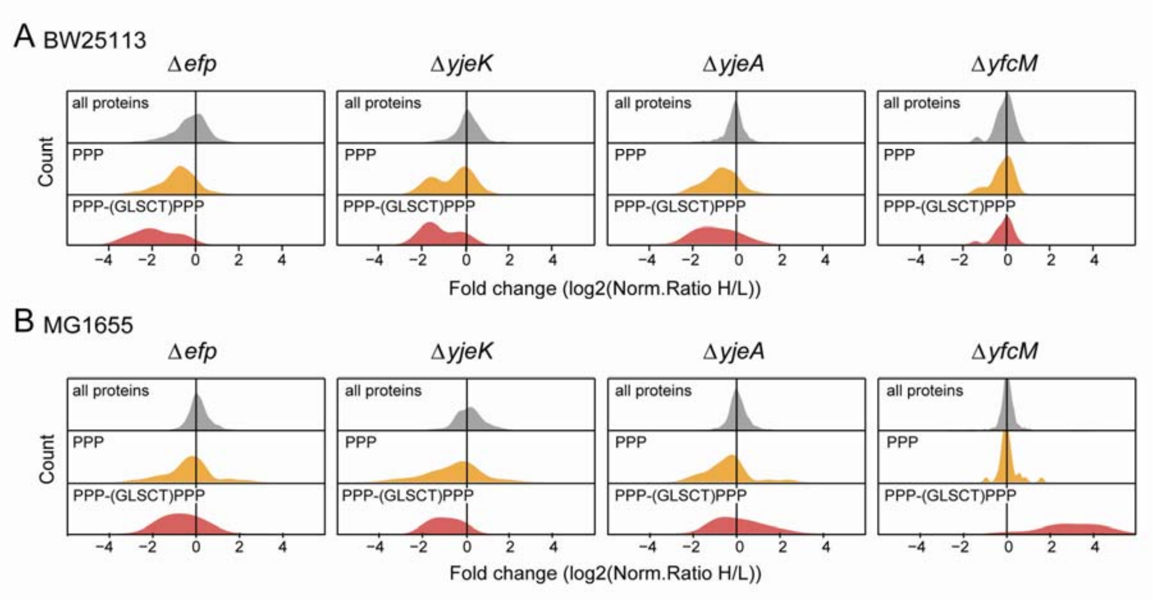

Supplementary Figure S3. G/L/S/C/TPPP motifs reduce ribosome stalling *in vivo*

Density plots displaying the distribution of all proteins (grey), PPP-containing proteins (orange), and PPP-containing proteins excluding weak stallers G/L/S/C/TPPP (red) from the Δ*efp*, Δ*yjeA (*Δ*epmA)*, Δ*yjeK (*Δ*epmB)* and Δ*yfcM* (Δ*epmC*) of (**A**) BW25113 and (**B**) MG1655 *E. coli* strains as a function of inverted normalized H/L ratios (log2-transformed). MG1655 SILAC data taken from (8). The enhanced production of production of PPP-(GLSCT) proteins for Δ*yfcM* in the MG1655 is not observed for the BW25113 data. Since the BW25113 data for Δ*yfcM* is based on the identification 16 proteins, whereas the MG1655 data is based on only 9 proteins, it is possible that the enhanced production of PPP-(GLSCT) proteins for Δ*yfcM* in the MG1655 is due to low signal to noise ratio.

Table S1: Plasmids, primers and strains used in this study

Plasmid	Feature / Construction comments	Source
p3LC-TL30	Translational CadC'-LacZ fusion (sequence encodes 30 amino acids of *cadC)*	(6)
pET21b	Cloning of nlpD and lepA for mutagenesis	Merck
p3LC-TL30-A3P	p3LC-TL30 + sequence encoding Ala-Pro-Pro-Pro	This study
p3LC-TL30-C3P	p3LC-TL30 + sequence encoding Cys-Pro-Pro-Pro	This study
p3LC-TL30-D3P	p3LC-TL30 + sequence encoding Asp-Pro-Pro-Pro	This study
p3LC-TL30-E3P	p3LC-TL30 + sequence encoding Glu-Pro-Pro-Pro	This study
p3LC-TL30-F3P	p3LC-TL30 + sequence encoding Phe-Pro-Pro-Pro	This study
p3LC-TL30-G3P	p3LC-TL30 + sequence encoding Gly-Pro-Pro-Pro	This study
p3LC-TL30-H3P	p3LC-TL30 + sequence encoding His-Pro-Pro-Pro	This study
p3LC-TL30-I3P	p3LC-TL30 + sequence encoding Ile-Pro-Pro-Pro	This study
p3LC-TL30-K3P	p3LC-TL30 + sequence encoding Lys-Pro-Pro-Pro	This study
p3LC-TL30-L3P	p3LC-TL30 + sequence encoding Leu-Pro-Pro-Pro	This study
p3LC-TL30-M3P	p3LC-TL30 + sequence encoding Met-Pro-Pro-Pro	This study
p3LC-TL30-N3P	p3LC-TL30 + sequence encoding Asn-Pro-Pro-Pro	This study
p3LC-TL30-P3P	p3LC-TL30 + sequence encoding Pro-Pro-Pro-Pro	This study
p3LC-TL30-Q3P	p3LC-TL30 + sequence encoding Gln-Pro-Pro-Pro	This study
p3LC-TL30-R3P	p3LC-TL30 + sequence encoding Arg-Pro-Pro-Pro	This study
p3LC-TL30-S3P	p3LC-TL30 + sequence encoding Ser-Pro-Pro-Pro	This study
p3LC-TL30-T3P	p3LC-TL30 + sequence encoding Thr-Pro-Pro-Pro	This study
p3LC-TL30-V3P	p3LC-TL30 + sequence encoding Val-Pro-Pro-Pro	This study
p3LC-TL30-W3P	p3LC-TL30 + sequence encoding Trp-Pro-Pro-Pro	This study
p3LC-TL30-Y3P	p3LC-TL30 + sequence encoding Tyr-Pro-Pro-Pro	This study
p3LC-nlpD	Translational-LacZ fusion encompassing the *cadC* promoter fused to nlpD aminoacids 25-54 (no X3P)	This study
p3LC-nlpD-A3P	p3LC-nlpD-Gly25–Ile53 + Ala-Pro-Pro-Pro	This study
p3LC-nlpD-C3P	p3LC-nlpD-Gly25 – Ile53 + Cys-Pro-Pro-Pro	This study
p3LC-nlpD-D3P	p3LC-nlpD-Gly25 – Ile53 + Asp-Pro-Pro-Pro	This study
p3LC-nlpD-E3P	p3LC-nlpD-Gly25 – Ile53 + Glu-Pro-Pro-Pro	This study
p3LC-nlpD-F3P	p3LC-nlpD-Gly25– Ile53 + Phe-Pro-Pro-Pro	This study
p3LC-nlpD-G3P	p3LC-nlpD-Gly25– Ile53 + Gly-Pro-Pro-Pro	This study
p3LC-nlpD-H3P	p3LC-nlpD-Gly25– Ile53 + His-Pro-Pro-Pro	This study
p3LC-nlpD-I3P	p3LC-nlpD-Gly25– Ile53 + Ile-Pro-Pro-Pro	This study
p3LC-nlpD-K3P	p3LC-nlpD-Gly25– Ile53 + Lys-Pro-Pro-Pro	This study
p3LC-nlpD-L3P	p3LC-nlpD-Gly25– Ile53 + Leu-Pro-Pro-Pro	This study
p3LC-nlpD-M3P	p3LC-nlpD-Gly25– Ile53 + Met-Pro-Pro-Pro	This study
p3LC-nlpD-N3P	p3LC-nlpD-Gly25– Ile53 + Asn-Pro-Pro-Pro	This study
p3LC-nlpD-P3P	p3LC-nlpD-Gly25 – Ile53 + Pro-Pro-Pro-Pro	This study
p3LC-nlpD-Q3P	p3LC-nlpD-Gly25 – Ile53 + Gln-Pro-Pro-Pro	This study
p3LC-nlpD-R3P	p3LC-nlpD-Gly25 – Ile53 + Arg-Pro-Pro-Pro	This study
p3LC-nlpD-S3P	p3LC-nlpD-Gly25 – Ile53 + Ser-Pro-Pro-Pro	This study
p3LC-nlpD-T3P	p3LC-nlpD-Gly25 – Ile53 + Thr-Pro-Pro-Pro	This study
p3LC-nlpD-V3P	p3LC-nlpD-Gly25 – Ile53 + Val-Pro-Pro-Pro	This study
p3LC-nlpD-W3P	p3LC-nlpD-Gly25 – Ile53 + Trp-Pro-Pro-Pro	This study
p3LC-nlpD-Y3P	p3LC-nlpD-Gly25 – Ile53 + Tyr-Pro-Pro-Pro	This study
pET21b-nlpD	NlpD wt: 45ANTNSGMLITPPP57	This study
pET21b-$_{5-1}$nlpD	$_{5-1}$NlpD : 45ANTNSLVRDIPPP57	This study
pET21b-$_{10-1}$nlpD	$_{10-1}$NlpD: 45DVLERLVRDIPPP57	This study
pET21b-$_{R5}$nlpD	$_{R5}$NlpD: 45ANTNSRMLITPPP57	This study
pET21b-$_{R4}$nlpD	$_{R4}$NlpD: 45ANTNSGRLITPPP57	This study
pET21b-$_{R3}$nlpD	$_{R3}$NlpD: 45ANTNSGMRITPPP57	This study
pET21b-$_{R2}$nlpD	$_{R2}$NlpD: 45ANTNSGMLRTPPP57	This study
pET21b-$_{R1}$nlpD	$_{R1}$NlpD: 45ANTNSGMLITRPP57	This study
pET21b-lepA	LepA wt: 171DVLERLVRDIPPP183	This study
pET21b-$_{5-1}$lepA	$_{5-1}$LepA: 171DVLERGMLITPPP183	This study
pET21b-$_{10-1}$lepA	$_{10-1}$LepA: 171ANTNSGMLITPPP183	This study
pET21b-toe-print	CATATGCATCATCATCATCATCACAAGAATATACGTAACTTTTCGATCATAGCTCACATTGAC<br>CACCTGCCGCCGCCGTTTACCTAATAAGAGCTCGGTAAATCGACGCTGTCTGACCGTATTAT<br>CCAGATCTGCGGTGGCCTGTCTGACCGTGAAATGGAGGCGCAGGTTCTC<br>Encoding: MHHHHHHKNIRNFSIIAHIDHLPPPFT-	This study

Primers	
FOR pET21b-nlpD	CTTTAAGAAGGAGATATACATATGATGCATCATCATCATCATCACAGCGCGGGAAGCCCAAAATTCACCG
REV pET21b-nlpD	CCGCAAGCTTGTCGACGGAGCTCTTATTATTATCGCTGCGGCAAATAACGC
FOR $_{5-1}$nlpD	GCGCCTGCAAATACTAATTCTCTGGTGCGCGACATT**CCGCCGCCG**AAAATGGGGACGACG
REV $_{5-1}$nlpD	CGTCGTCCCCATTTTCGGCGGCGGAATGTCGCGCACCAGAGAATTAGTATTTGCAGGCGC
FOR $_{10-1}$nlpD	GCTCCGTTAATGGCAATGCGCCTGACGTTCTCGAACGTCTGGTGCGCGACATT**CCGCCGCCG**AAAATGGGGACGACG
REV $_{10-1}$nlpD	CGTCGTCCCCATTTTCGGCGGCGGAATGTCGCGCACCAGACGTTCGAGAACGTCAGGCGCATTGCCATTAACGGAGC
FOR $_{R5}$nlpD	GGCAATGCGCCTGCAAATACTAATTCTAGAATGTTGATTACG**CCGCCGCCG**AAAATGGGG
REV $_{R5}$nlpD	CCCCATTTTCGGCGGCGGCGTAATCAACATTCTAGAATTAGTATTTGCAGGCGCATTGCC
FOR $_{R4}$nlpD	GCGCCTGCAAATACTAATTCTGGTAGATTGATTACG**CCGCCGCCG**AAAATGGGG
REV $_{R4}$nlpD	CCCCATTTTCGGCGGCGGCGTAATCAATCTACCAGAATTAGTATTTGCAGGCGC
FOR $_{R3}$nlpD	GCGCCTGCAAATACTAATTCTGGTATGAGAATTACG**CCGCCGCCG**AAAATGGGGACGACG
REV $_{R3}$nlpD	CGTCGTCCCCATTTTCGGCGGCGGCGTAATTCTCATACCAGAATTAGTATTTGCAGGCGC
FOR $_{R2}$nlpD	GCGCCTGCAAATACTAATTCTGGTATGTTGAGAACG**CCGCCGCCG**AAAATGGGGACGACG
REV $_{R2}$nlpD	CGTCGTCCCCATTTTCGGCGGCGGCGTTCTCAACATACCAGAATTAGTATTTGCAGGCGC
FOR $_{R1}$nlpD	GCAAATACTAATTCTGGTATGTTGATT**AGACCGCCGCCG**AAAATGGGGACGACGTCG
REV $_{R1}$nlpD	CGACGTCGTCCCCATTTTCGGCGGCGGTCTAATCAACATACCAGAATTAGTATTTGC
FOR $_{C1}$nlpD	GCAAATACTAATTCTGGTATGTTGATTTGC**CCGCCGCCG**AAAATGGGGACGACGTCG
REV $_{C1}$nlpD	CGACGTCGTCCCCATTTTCGGCGGCGGGCAAATCAACATACCAGAATTAGTATTTGC
FOR $_{A1}$nlpD	GCAAATACTAATTCTGGTATGTTGATT**GCGCCGCCGCCGA**AAATGGGGACGACGTCG
REV $_{A1}$nlpD	CGACGTCGTCCCCATTTTCGGCGGCGGCGCAATCAACATACCAGAATTAGTATTTGC
FOR $_{F1}$nlpD	GCAAATACTAATTCTGGTATGTTGATT**TTCCCGCCGCCG**AAAATGGGGACGACGTCG
REV $_{F1}$nlpD	CGACGTCGTCCCCATTTTCGGCGGCGGGAAAATCAACATACCAGAATTAGTATTTGC
FOR $_{E1}$nlpD	GCAAATACTAATTCTGGTATGTTGATT**GAACCGCCGCCG**AAAATGGGGACGACGTCG
REV $_{E1}$nlpD	CGACGTCGTCCCCATTTTCGGCGGCGGTTCAATCAACATACCAGAATTAGTATTTGC

FOR $_{Q1}$nlpD	GCAAATACTAATTCTGGTATGTTGATT**CAGCCGCCGCCG**AAAATGGGGACGACGTCG
REV $_{Q1}$nlpD	CGACGTCGTCCCCATTTTCGGCGGCGGCTGAATCAACATACCAGAATTAGTATTTGC
FOR $_{L1}$nlpD	GCAAATACTAATTCTGGTATGTTGATT**CTGCCGCCGCCG**AAAATGGGGACGACGTCG
REV $_{L1}$nlpD	CGACGTCGTCCCCATTTTCGGCGGCGGCAGAATCAACATACCAGAATTAGTATTTGC
FOR $_{I1}$nlpD	GCAAATACTAATTCTGGTATGTTGATT**ATTCCGCCGCCG**AAAATGGGGACGACGTCG
REV $_{I1}$nlpD	CGACGTCGTCCCCATTTTCGGCGGCGGAATAATCAACATACCAGAATTAGTATTTGC
FOR $_{S1}$nlpD	GCAAATACTAATTCTGGTATGTTGATT**AGCCCGCCGCCG**AAAATGGGGACGACGTCG
REV $_{S1}$nlpD	CGACGTCGTCCCCATTTTCGGCGGCGGGCTAATCAACATACCAGAATTAGTATTTGC
FOR pET21b-lepA	CTTTAAGAAGGAGATATACATATGCATCATCATCATCATCACAAGAATATACGTAACTTTTCGATCATAGC
REV pET21b-lepA	CCGCAAGCTTGTCGACGGAGCTCTTATTTGTTGTCTTTGCCGACGTGC
FOR $_{5\text{-}1}$lepA	GGTGTGCAGGACGTTCTCGAACGTGGTATGTTGATTACG**CCGCCGCCG**GAAGGCGATCCGGAAGG
REV $_{5\text{-}1}$lepA	CCTTCCGGATCGCCTTCCGGCGGCGGCGTAATCAACATACCACGTTCGAGAACGTCCTGCACACC
FOR $_{10\text{-}1}$lepA	CGAAAACCGGCGTTGGTGTGCAGGCAAATACTAATTCTGGTATGTTGATTACG**CCGCCGCCG**GAAGGCGATCCGGAAGG
REV $_{10\text{-}1}$lepA	CCTTCCGGATCGCCTTCCGGCGGCGGCGTAATCAACATACCAGAATTAGTATTTGCCTGCACACCAACGCCGGTTTTCG
FOR Agp	TAATACGACTCACTATAGGGTTTAACTTTAAGAAGGAGATATACCATGAACAAAACGCTAATCGCCGCAGC
REV Agp	CTAGTTATTGCTCAGCGGTTTATTTCACCGCTTCATTCAACACGC
FOR NudC	AATAATACGACTCACTATAGGGTTTAACTTTAAGAAGGAGATATACCATGGATCGTATAATTGAAAAATTAG
REV NudC	TTCTAGTTATTGCTCAGCGGTTTACTCATACTCTGCCCGACACATCG
FOR YcgL	AATAATACGACTCACTATAGGGTTTAACTTTAAGAAGGAGATATACCATGTTTTGTGTGATTTATCGAAGC
REV YcgL	TTCTAGTTATTGCTCAGCGGTTTATTTGTTAGTGTCGTCTGTTTTCTG
FOR ClsA	TAATACGACTCACTATAGGGTTTAACTTTAAGAAGGAGATATACCATGACAACCGTTTATACGTTGGTGAG
REV ClsA	CTAGTTATTGCTCAGCGGTTTACAGCAACGGACTGAAGAAGTAAAACAGTCGC
REV NlpD toeprint	TGGCTGGATTTGCGGTTGTTGAG
REV short PPP template toeprint	GAGAACCTGCGCCTCCATTTCACGGTC
PciI-NlpD-A3P-Rev	GTACATGTGCGGCGGCGGCGCAATCAACATACCAGA
PciI-NlpD-C3P-Rev	GTACATGTGCGGCGGCGGGCAAATCAACATACCAGA
PciI-NlpD-D3P-Rev	GTACATGTGCGGCGGCGGATCAATCAACATACCAGA
PciI-NlpD-E3P-Rev	GTACATGTGCGGCGGCGGTTCAATCAACATACCAGA
PciI-NlpD-F3P-Rev	GTACATGTGCGGCGGCGGAAAAATCAACATACCAGA
PciI-NlpD-G3P-Rev	GTACATGTGCGGCGGCGGGCCAATCAACATACCAGA
PciI-NlpD-H3P-Rev	GTACATGTGCGGCGGCGGATGAATCAACATACCAGA
PciI-NlpD-I3P-Rev	GTACATGTGCGGCGGCGGAATAATCAACATACCAGA
PciI-NlpD-K3P-Rev	GTACATGTGCGGCGGCGGTTTAATCAACATACCAGA
PciI-NlpD-L3P-Rev	GTACATGTGCGGCGGCGGCAGAATCAACATACCAGA
PciI-NlpD-M3P-Rev	GTACATGTGCGGCGGCGGCATAATCAACATACCAGA
PciI-NlpD-N3P-Rev	GTACATGTGCGGCGGCGGGTTAATCAACATACCAGA
PciI-NlpD-P3P-Rev	GTACATGTGCGGCGGCGGCGGAATCAACATACCAGA
PciI-NlpD-Q3P-Rev	GTACATGTGCGGCGGCGGCTGAATCAACATACCAGA
PciI-NlpD-R3P-Rev	GTACATGTGCGGCGGCGGGCGAATCAACATACCAGA
PciI-NlpD-S3P-Rev	GTACATGTGCGGCGGCGGGCTAATCAACATACCAGA
PciI-NlpD-T3P-Rev	GTACATGTGCGGCGGCGGGGTAATCAACATACCAGA
PciI-NlpD-V3P-Rev	GTACATGTGCGGCGGCGGCACAATCAACATACCAGA
PciI-NlpD-W3P-Rev	GTACATGTGCGGCGGCGGCCAAATCAACATACCAGA
PciI-NlpD-Y3P-Rev	GTACATGTGCGGCGGCGGATAAATCAACATACCAGA
PciI-TF030-CadC-A3P-Rev	GTACATGTGCGGCGGCGGCGCCTCAAGGGTAAGTTGACGCCCATTGCG
PciI-TF030-CadC-C3P-Rev	GTACATGTGCGGCGGCGGGCACTCAAGGGTAAGTTGACGCCCATTGCG
PciI-TF030-CadC-D3P-Rev	GTACATGTGCGGCGGCGGATCCTCAAGGGTAAGTTGACGCCCATTGCG
PciI-TF030-CadC-E3P-Rev	GTACATGTGCGGCGGCGGTTCCTCAAGGGTAAGTTGACGCCCATTGCG
PciI-TF030-CadC-F3P-Rev	GTACATGTGCGGCGGCGGAAACTCAAGGGTAAGTTGACGCCCATTGCG
PciI-TF030-CadC-G3P-Rev	GTACATGTGCGGCGGCGGGCCCTCAAGGGTAAGTTGACGCCCATTGCG
PciI-TF030-CadC-H3P-Rev	GTACATGTGCGGCGGCGGATGCTCAAGGGTAAGTTGACGCCCATTGCG
PciI-TF030-CadC-I3P-Rev	GTACATGTGCGGCGGCGGAATCTCAAGGGTAAGTTGACGCCCATTGCG
PciI-TF030-CadC-K3P-Rev	GTACATGTGCGGCGGCGGTTTCTCAAGGGTAAGTTGACGCCCATTGCG
PciI-TF030-CadC-L3P-Rev	GTACATGTGCGGCGGCGGCAGCTCAAGGGTAAGTTGACGCCCATTGCG
PciI-TF030-CadC-M3P-Rev	GTACATGTGCGGCGGCGGCATCTCAAGGGTAAGTTGACGCCCATTGCG
PciI-TF030-CadC-N3P-Rev	GTACATGTGCGGCGGCGGGTTCTCAAGGGTAAGTTGACGCCCATTGCG
PciI-TF030-CadC-P3P-Rev	GTACATGTGCGGCGGCGGCGGCTCAAGGGTAAGTTGACGCCCATTGCG
PciI-TF030-CadC-Q3P-Rev	GTACATGTGCGGCGGCGGCTGCTCAAGGGTAAGTTGACGCCCATTGCG
PciI-TF030-CadC-R3P-Rev	GTACATGTGCGGCGGCGGGCGCTCAAGGGTAAGTTGACGCCCATTGCG
PciI-TF030-CadC-S3P-Rev	GTACATGTGCGGCGGCGGGCTCTCAAGGGTAAGTTGACGCCCATTGCG
PciI-TF030-CadC-T3P-Rev	GTACATGTGCGGCGGCGGGGTCTCAAGGGTAAGTTGACGCCCATTGCG
PciI-TF030-CadC-V3P-Rev	GTACATGTGCGGCGGCGGCACCTCAAGGGTAAGTTGACGCCCATTGCG
PciI-TF030-CadC-W3P-Rev	GTACATGTGCGGCGGCGGCCACTCAAGGGTAAGTTGACGCCCATTGCG
PciI-TF030-CadC-Y3P-Rev	GTACATGTGCGGCGGCGGATACTCAAGGGTAAGTTGACGCCCATTGCG

Strain	Feature / Construction comments	Source
DH5α	F^- Φ80*lac*ZΔM15 Δ(*lac*ZYA-*arg*F) U169 *rec*A1 *end*A1 *hsd*R17 (rK^-, mK^+) *pho*A *sup*E44 λ^- *thi*-1 *gyr*A96 *rel*A1	Promega
BW25113	Δ*(araD-araB)567*, Δ*lacZ4787*(::rrnB-3), $lambda^-$, *rph-1*, Δ*(rhaD-rhaB)568*, *hsdR514*	(15)
Δ*efp*	BW25113, Δ*efp, ΔargA, ΔlysA*	This study
Δ*yjeA (*Δ*epmA)*	BW25113, Δ*yjeA, ΔargA, ΔlysA*	This study
Δ*yjeK (*Δ*epmB)*	BW25113, Δ*yjeK, ΔargA, ΔlysA*	This study
Δ*yfcM (*Δ*epmC)*	BW25113, ΔyfcM*, ΔargA, ΔlysA*	This study
